# Enthusiasm for Introducing and Integrating HIV Self-Testing but Doubts About Users: A Baseline Qualitative Analysis of Key Stakeholders' Attitudes and Perceptions in Côte d'Ivoire, Mali and Senegal

**DOI:** 10.3389/fpubh.2021.653481

**Published:** 2021-10-18

**Authors:** Odette Ky-Zerbo, Alice Desclaux, Alexis Brou Kouadio, Nicolas Rouveau, Anthony Vautier, Souleymane Sow, Sidi Cheick Camara, Sokhna Boye, Dolorès Pourette, Younoussa Sidibé, Mathieu Maheu-Giroux, Joseph Larmarange

**Affiliations:** ^1^TransVIHMI, Université de Montpellier, IRD, INSERM, Montpellier, France; ^2^TransVIHMI, IRD, INSERM, University of Montpellier, Center Régional de Recherche et de Formation au VIH et Maladies Associées de Fann, Dakar, Senegal; ^3^Département de Sociologie, Institut d'ethnosociologie (IES), Université Félix Houphouët Boigny de Cocody, Abidjan, Côte d'Ivoire; ^4^Ceped, IRD, Université de Paris, Inserm, Paris, France; ^5^Solidarité Thérapeutique et Initiatives Pour la Santé, Dakar, Senegal; ^6^Center Régional de Recherche et de Formation à la Prise en Charge Clinique de Fann (CRCF), Dakar, Senegal; ^7^Département Santé, Institut Malien de Recherche en Sciences Sociales (IMRSS), Bamako, Mali; ^8^Solidarité Thérapeutique et Initiatives pour la Santé, Bamako, Mali; ^9^Department of Epidemiology, Biostatistics, and Occupational Health, School of Population and Global Health, McGill University, Montréal, QC, Canada

**Keywords:** HIV self-testing, key population, perceptions, stakeholders, West Africa, ATLAS

## Abstract

Since 2019, the ATLAS project, coordinated by Solthis in collaboration with national AIDS programs, has introduced, promoted and delivered HIV self-testing (HIVST) in Côte d'Ivoire, Mali and Senegal. Several delivery channels have been defined, including key populations: men who have sex with men, female sex workers and people who use injectable drugs. At project initiation, a qualitative study analyzing the perceptions and attitudes of key stakeholders regarding the introduction of HIVST in their countries and its integration with other testing strategies for key populations was conducted. The study was conducted from September to November 2019 within 3 months of the initiation of HIVST distribution. Individual interviews were conducted with 60 key informants involved in the project or in providing support and care to key populations: members of health ministries, national AIDS councils, international organizations, national and international non-governmental organizations, and peer educators. Semi structured interviews were recorded, translated when necessary, and transcribed. Data were coded using Dedoose© software for thematic analyses. We found that stakeholders' perceptions and attitudes are favorable to the introduction and integration of HIVST for several reasons. Some of these reasons are held in common, and some are specific to each key population and country. Overall, HIVST is considered able to reduce stigma; preserve anonymity and confidentiality; reach key populations that do not access testing *via* the usual strategies; remove spatial barriers; save time for users and providers; and empower users with autonomy and responsibility. It is non-invasive and easy to use. However, participants also fear, question and doubt users' autonomy regarding their ability to use HIVST kits correctly; to ensure quality secondary distribution; to accept a reactive test result; and to use confirmation testing and care services. For stakeholders, HIVST is considered an attractive strategy to improve access to HIV testing for key populations. Their doubts about users' capacities could be a matter for reflective communication with stakeholders and local adaptation before the implementation of HIVST in new countries. Those perceptions may reflect the West African HIV situation through the emphasis they place on the roles of HIV stigma and disclosure in HIVST efficiency.

## Introduction

To eliminate the HIV epidemic by 2030, the Joint United Nations Programme for HIV/AIDS (UNAIDS) has set targets of 95% diagnosis coverage by 2030 (along with 95% treatment among diagnosed people living with HIV–PLHIV- and 95% viral suppression among those on treatment) (1). Estimates at the end of 2019 showed rates of 81-82-88, and disparities were observed between regions and countries. The corresponding rates were only 68-58-45 in West and Central Africa (2). The last published data confirmed that the rates of knowledge of HIV status by PLHIV are much lower in the countries of West and Central Africa, than those from Eastern and Southern Africa (3).

The underachievement of the first rate can be explained by social factors that negatively influence HIV testing services (HTS) uptake in sub-Saharan Africa. They include fear of HIV, which is a barrier to testing uptake (4), low perceptions of exposure to HIV risk, which can positively (5), or negatively (6–8) influence adherence to testing; and HIV-related stigma and discrimination, which are the main barriers to HTS utilization (7–11). Stigma is reported to be more pronounced in West Africa than in Eastern and Southern Africa (12). The main barrier to couple testing remains the fear of negative consequences, which negatively influences the disclosure of HIV results between sexual partners (13–15).

HIV epidemics in West Africa disproportionately affect members of key populations and their partners: female sex workers (FSW), men who have sex with men (MSM), people who use injectable drugs (PWuIDs), transgender people and prisoners (2). These populations have important unmet HIV prevention needs in this region, where they are subject to intense social or structural stigmatization. Such stigma reduces their ability to seek, access, and use health services, including HTS (16–18).

These social barriers need to be removed to improve HTS access and uptake while protecting the privacy and confidentiality of HIV test results. Overall, confidentiality has been identified as a critical factor for HTS uptake (7, 8, 16). HIV self-testing (HIVST) is offering such a guarantee. This modality is defined by the World Health Organization (WHO) as a “*process in which an individual collects a specimen (saliva or blood) using a simple, rapid HIV test, performs a test, and then interprets the result when and where he/she wants it*” (19). HIVST on its own does not necessarily provide a definitive diagnosis, however. People with a reactive (positive) result must confirm the result through facility-based testing or with a trained professional. Those with a non-reactive (negative) result do not need a confirmatory test unless they have been recently exposed to the virus or are in the initiation phase of pre-exposure prophylaxis. However, a negative test result is an opportunity to connect with other prevention services. WHO does not recommend HIVST for PLHIV on antiretroviral treatment, as they risk obtaining false negatives results. Since November 2019, the WHO has recommended that HIVST be offered by health facilities as part of HTS (19).

This innovative strategy has been implemented in several regions since 2010, and the results of studies in sub-Saharan Africa, mainly conducted in Southern and Eastern Africa, have shown variable but generally high acceptability rates (20). Among the general population, acceptability rates are above 94% in Kenya and Malawi (10, 21). Studies in Eastern and Southern Africa have also found that HIVST is acceptable among key populations and is effective in identifying PLHIV who are unaware of their status, both among MSM and FSW (21–23). However, HIVST is poorly documented in francophone West African countries, where the national HIV prevalence is much lower than in Eastern and Southern Africa.

Coordinated by Solthis, an international non-governmental organization (NGO), and the Institut de Recherche pour le Développement (IRD), the ATLAS program (*AutoTest VIH, Libre d'Accéder à la connaissance de son Statut*) aims to promote and distribute HIVST in three West African countries (Côte d'Ivoire, Mali, and Senegal) from 2019 to 2021, in close collaboration with national AIDS councils, civil society organizations and key population communities. Considering West African countries' HIV epidemiology, the main focus of ATLAS is key populations (FSW, MSM, and PWuIDs) and their sexual partners, peers and clients; sexually transmitted infection patients and their partners; and the partners of PLHIV. An oral HIVST OraQuick HIV Self-Test® (OraSure Technologies, LLC Bethlehem) will be used as it is pre-qualified by WHO and has been validated by the three countries of intervention. To facilitate HIVST uptake and promote the link to confirmation testing and care services, locally adapted brochures describing HIVST steps in addition to the manufacturer's instructions for use and videos in French and other national languages have been developed. Existing free HIV hotlines in each country were reinforced and their managers trained in HIVST.

In parallel with the implementation, ATLAS includes a research component and has run several qualitative and quantitative studies; in particular, a qualitative study conducted at program implementation has documented and analyzed HTS stakeholders' and key actors' perceptions and attitudes regarding the introduction of HIVST in Côte d'Ivoire, Mali and Senegal and its integration as a strategy for key populations.

## Materials and Methods

This qualitative study was conducted in Côte d'Ivoire, Mali and Senegal from September to November 2019, within 3 months of the beginning of HIVST delivery activities. In each country, one urban and one rural cities were selected by the teams of ATLAS program who have a good knowledge of the stakeholders at the national level: Abidjan and Mafere in Côte d'Ivoire, Bamako and Kati in Mali, Dakar and Thies in Senegal. These sites were also the implementations ones. Since it is not a representative study, the study results could be useful.

### Participants

Mapping of HTS stakeholders was carried out with the local ATLAS implementation teams to identify study participants, who received an invitation letter from ATLAS program, inviting them to take part to a study on HIVST perceptions. They were selected because of their good knowledge of key populations and their relationship to HIV and health. All chosen participants were fully involved in the coordination or delivery of HTS to key populations. On this background, they have been identified on a personal title or by their respective structures. Thus, the research team managed a meeting with them for the interview.

### Data Collection

Individual face-to-face interviews were conducted by two trained interviewers: the field research coordinator (MPH, PhD), and a local research assistant in each country (PhD candidates (Sociology) in Côte d'Ivoire and Senegal, Master (sociology) in Mali. A semi structured interview guide was used. Interviews took place in participants' offices, community life spaces (public services, NGOs/associations) or homes. Four participants were not available for face-to-face meetings and were interviewed by telephone (one NGO responsible in Côte d'Ivoire, one health provider and one from the national AIDS program in Senegal, one health provider in Mali). Three participants from the urban area were not interviewed because they were traveling for work (one from the Ministry of health in Mali, one NGO responsible in Côte d'Ivoire) or was on vacation (one in Côte d'Ivoire). They were not replaced because data saturation is observed in each country by the field research coordinator. The interviews, which lasted from 45 to 60 min, covered participants' attitudes and perceptions on (I) opportunities, difficulties and obstacles to the introduction of HIVST and HIVST support tools in the three countries' health system and community-based organizations; (II) difficulties and obstacles linked to secondary distribution; (III) specific difficulties and obstacles for each key population; (IV) support tools for users and links to confirmation testing (advice, hotline, and support tools); and (V) adjustments and recommendations for key populations. The identification of the topics was based on the literature contents at this time, the authors' knowledge on the study context and the needs of the ATLAS project. For this paper, the analyses focuses on data related to topics I to III and V.

### Data Treatment and Analysis

Interviews were audio-recorded, translated where necessary, transcribed by each country research assistant, and anonymized to ensure confidentiality. Transcripts were proofread and corrected by the field research coordinator. She designed the coding framework on the basis of the respondents' discourses. Then the transcripts were coded by two researchers involved in data collection (the field research coordinator and one research assistant), who were familiarized with the research subject. First, three transcripts were coded by the two researchers. This process allowed comparison, discussion, correction and agreement on the framework between them. They coded the transcripts, using Dedoose software (Dedoose.com). A coding report was exported to Word, and a thematic analysis was then carried out code by code by the two researchers, followed by a cross-analysis. Three topics were selected for this analysis: driving factors, Concerns & doubts and the respondents' suggestions. Sub-themes that flow from each of these topics were identified from the respondents' discourses for analysis.

### Ethical Considerations

Both the research protocol and the data collection tools have been approved by the WHO and the countries' ethics committees: WHO Ethical Research Committee (2019, August 7th, reference: ERC 0003181); National Ethics Committee for Life Sciences and Health of Côte d'Ivoire (2019, May 28th, reference: 049-19/MSHP/CNESVS-kp); Ethics Committee of the Faculty of Medicine and Pharmacy of the University of Bamako, Mali (2019, August 14th, reference: 2019/88/CE/FMPOS); and the National Ethics Committee for Health Research of Senegal (2019, July 26th, protocol SEN19/32).

The research information sheet was read to respondents before each interview. For face-to-face interviews, all individuals signed a written consent form covering their participation and the audio recording. A copy of the information sheets and signed consents were given to the respondents. Oral consent was obtained from respondents who were interviewed by telephone. Interviews took place in private places, chosen by the respondents, between researchers and respondents only. No name was taken. Interviews were transcribed by the research assistant who participated to the interview, and data were anonymized before codification and analysis.

## Results

A total of 60 individuals were interviewed (19 in Côte d'Ivoire, 20 in Mali and 21 in Senegal) through 57 interviews (3 interviews were conducted with two participants simultaneously; see Table 1). One-third were female (20/60), and most of them lived in the main cities (47/60). Among all the participants, 15 were from public services (national AIDS programs, ministries of health), 6 were from international organizations (United Nations system, research institute), and 39 were from national or international NGOs. Of these, 11 were MSM, FSW or PWuID peer educators involved in HIV prevention and testing services for key populations.

**Table 1:** Participants' characteristics.

**Description**	**Country**			**Total** **(*N =* 60)**
	**Côte d'Ivoire** **(*N =* 19)**	**Mali** **(*N =* 20)**	**Senegal** **(*N =* 21)**	
**Gender**				
Female	9	3	8	20
Male	10	17	13	40
**Location**				
Urban	16	17	14	47
Other localities	3	3	7	13
**Structure**				
NGO/association	13	17	9	39
Governmental offices	3	1	11	15
International organizations	3	2	1	6
**Role in HTS**				
Peer educator/mediator	5	3	3	11
Other responsibilities	14	17	18	49

Three mains topics emerge of the data analysis: factors driving the introduction of HIVST in these countries; the stakeholders' concerns, fears and doubts; and their suggestions for the implementation of the project in their contexts. Each of these themes is outlined by sub-themes.

### Factors Driving the Introduction of HIVST in These Countries

From respondents' discourses, there are many motivations for HIVST introduction in their countries, which could be classified in categories: less stigma, testing hard to reach key population, removing spatial barriers of testing, an alternative tool for usual strategies testing refusers, empowerment of key population and strengthening health and Community system.

In Côte d'Ivoire, Mali and Senegal, HIVST raised hopes among the stakeholders interviewed, as it was expected to improve knowledge of HIV status among key populations. All participants had a favorable attitude toward its integration into national systems as a strategy for key populations. In their view, its advantage was that it removed the obstacles to testing by diversifying offerings and encouraging innovative strategies to achieve high diagnosis coverage. These favorable attitudes were based on interviewees' positive perceptions of HIVST at several levels.

#### HIVST May Minimize Stigma and Protect Anonymity and Confidentiality

The most crucial advantage of HIVST perceived by most respondents in all three countries was its protection of anonymity and confidentiality. In all the countries, but especially in Senegal, respondents stated that key populations, especially MSM and PWuID, fear the stigma they may face in community organizations or in health facilities because their behavior or identity is outside of accepted social norms. Stigma impedes their uptake of HTS. An HIVST could help mitigate these barriers because it is anonymous. Members of key populations would not have to fear being identified by providers or other users of these services, as their identity cannot be recorded when using HIVST.

(Usually), upon going to the facilities, people are registered, as they have come to be tested. Anonymity is, therefore, immediately lost (Medical doctor, key populations care provider, NGO, Mali).

We know many (PWuID) on the ground, but we have difficulty getting them to come to (name of the structure)… The more stigmatized they are, the more they stigmatize themselves (Medical doctor, PWuID care, governmental office, Senegal).

Also, through HIVST, it may be possible to better protect the sexual networks of members of key populations, as they themselves interact with their partners for secondary distribution without the intervention of providers.

*In the case of the usual rapid test, the peer educator must be present, and assistance is needed. In contrast, in the case of HIVST, people are free to reach their hidden partners. There is much more confidentiality and discretion (Program officer, NGO, Senegal)*.

Finally, usual outreach strategies can help people avoid having to visit facilities, according to respondents in all three countries, some key populations, especially MSM and PWuID, are concerned that HTS providers, especially peers, may know or discover their HIV test results. The HIVST could respond to their need for a higher level of confidentiality. The testing process can be conducted in private, without the involvement of a third party, since the testing, results, care and treatment sites are known only to the user. This tool may encourage people to learn their HIV status and thus improve testing uptake.

When we take the key populations… When the peer comes, they refuse because maybe there is this lack of confidentiality: will the peer not disclose my result and everything. If they are offered a self-test, they will quietly go home and do the test (National stakeholder, governmental office, Côte d'Ivoire).

So when we take the specific case of key populations, they are much more afraid of their peers than of the community… because it's a closed environment, everyone knows each other, so there is a real fear that the status will be known in the environment and the risk is that they will no longer have sexual partners (Medical doctor, key population care facility stakeholder, Mali).

#### HIVST May Help Reach Key Populations That Usual Strategies Cannot Reach

The main advantage of HIVST, as expressed by participants from all three countries, is the opportunities it affords for secondary distribution. According to them, it may allow the detection of undiagnosed PLHIV, particularly FSW and MSM, who cannot be reached through the usual strategies for various reasons: (1) they do not identify themselves as belonging to any key populations; (2) they refuse to visit governmental facilities or community-based organizations because of stigma or self-stigmatization; (3) they do not present themselves as belonging to key populations or are hiding; and (4) they are reluctant to get tested through the usual strategies. Specifically, concerning FSW, such people may include their partners and clients and “clandestine FSW,” who often refuse usual HIV testing for fear that their results will be made known to providers or to their peers.

A FSW who comes, if she agrees to do the test you will find that she has her sexual partner… But he refuses to be tested. We explain it to her, we give her the kit, and then she can go and give (it to) the partner (NGO responsible, Mali).

Hard-to-reach MSM mentioned by the respondents included those with high social status, who are older, who are married (to women) or who have certain social or professional responsibilities.

There are many tops (insertive sexual role), but they don't think of themselves as MSM. They are men, they have their girlfriend and they always come to us, they date (have sex) with us. They really love us; they are always with us. And if there's anything else, they do it with their girlfriends. In any case, they don't consider themselves MSM (MSM peer educator, Senegal).

Finally, according to some respondents, providing HIVST could be an opportunity to facilitate index testing among key populations.

#### HIVST May Remove Spatial Barriers to HTS and Save Time

Participants in all countries found that HIVST prevents key populations from needing to go to health facilities or community-based organizations, i.e., it saves time and reduces travel costs.

When they want to do HIV testing in a health facility, they must go there. They spend money to go, they spend money to come back and they use their time too; but with HIVST, they can do it with their FSW friend (field coordinator, NGO, Mali).

In addition to saving transport time, HIVST eliminates time spent in health facilities or community-based organizations waiting to be tested or to receive results. From the perspective of the participants from Côte d'Ivoire and Senegal, this advantage seemed to be more beneficial to PWuID.

As for testing activities I participated in, almost an hour was needed to find out one's status. Time is precious for a population like PWuID because they are constantly looking for money to solve their problem. If you keep such a person for more than 2 h, it may bring trouble (Peer educator, PWuID, Senegal).

For FSW, according to some respondents, HIV testing through outreach strategies has limitations. Its inconvenience for FSW is their lack of availability at sex work sites. HIVST introduction could help to mitigate this problem, as long as FSW could take the kits home and test themselves later.

Specifically, for FSW, when you arrive at the venues, you know they are looking for clients, they do not necessarily have the time to test. Providing them with self-testing kits will save them time and also prevent them from losing clients who are waiting for them (International NGO Responsible, Senegal).

#### HIVST May Be an Alternative Tool for HIV Testing Strategies

According to HIVST providers from community-based organizations, especially in Mali and Côte d'Ivoire, HIV testing refusals are not uncommon during outreach activities. Having an alternative solution, such as HIVST, for key populations who may decline conventional testing could boost the morale of peer educators because they will be less helpless in such situations.

We'd come back and it was not too quiet because we'd come back and there were other people who refused the classic test. It's rare to go out (in the field) and you really don't have anyone who has never had somebody refuse the classic test. So when you have an alternative for that… (Medical doctor, field coordinator, Mali).

Beyond providing an alternative when faced with refusals, HIVST could be used to compensate for the lack of HTS provision to key populations when certain social situations do not allow in-person meetings, as reported by a participant from Senegal. He referred to the national context at the time of data collection, where media and public opinion were overtly hostile to MSM, preventing them from accessing health facilities.

#### HIVST May Empower Users by Giving Them Autonomy and Responsibility

The interviews with participants also revealed their perception that HIVST empowers key populations by making them responsible for their own health because they are free to choose where and when to carry out HIVST, without any pressure from HTS providers.

There is autonomy, i.e., I'm not the one who's going to say OK, we'll do it now; you're autonomous, you have your test, if it's in the evening, it's daytime, tomorrow, the day after tomorrow, so you're independent (Medical doctor, field coordinator, Mali).

In addition to choosing the place and time of testing, key populations are fully empowered because they perform the test themselves, interpret the results, and then choose a care facility for confirmation, independent of any community or health provider. In Senegal and Côte d'Ivoire, this empowerment would give members of key populations a role as HTS actors in the sense that, in the context of secondary distribution, they could raise awareness among other members of their entourage and offer HIVST kits.

It allows them to participate as well, since they have to send the HIVST to their partners and others who are not there. So, they can be at the heart of the project, participate in the project as well (Program manager, NGO, Côte d'Ivoire).

Stakeholders stated that HIVST is a solution for key populations, particularly certain FSW whose partners do not allow them to visit HIV testing facilities. It allows them to learn their status independently of these partners.

#### HIVST May Help Strengthen the Community and Health System

In all three countries, according to some participants, the introduction of HIVST is a way to strengthen community-based organizations, a tool that will enable them to extend testing strategies and reach the “first 90.” They state that it will also provide an alternative in the eventuality that key populations decline testing, notably for reasons of confidentiality. In Mali and Senegal, the economic advantages of this strategy were pointed out, as HIVST does not require mobilization of a full testing team for outreach activities. Finally, HIVST is safer, as outreach personnel avoid contact with body fluids to which they may be exposed in the context of their HIV testing activities.

In Côte d'Ivoire and Senegal, participants noted that HIVST does not require qualified personnel, and its introduction could help relieve the pressure on health facilities if the cases they receive are only reactive cases requiring confirmation. This strategy could help to address the lack of skilled human resources in health facilities and allow health providers to delegate HIV testing to others and thereby have more time for care.

If I have to do 20 tests a day, I can't get away with it when I have other things to do. Therefore, it frees up time to deal with other diseases, other patients (Medical doctor, PLHIV care, Côte d'Ivoire).

#### Oral HIVST May Be Appreciated for Being Non-invasive and Easy to Use

Informants, especially those from Mali and Senegal, mentioned the test's oral nature, which may facilitate the uptake of HIVST by key populations, especially MSM and PWuID. Some people may decline traditional testing because of the collection of blood, which can be painful and exposes users to the sight of blood (Notably, in West Africa, most PWuID smoke the drug; few inject it).

As far as PWuID are concerned, they are a bit resistant to taking blood too… Some refused to have their blood taken for testing. So having another strategy that doesn't use blood, for me, it's something that will really solve an important gap in this system (HIVST provider, public office, Senegal).

The availability of an oral test may allow HTS to be offered to key populations who would refuse the test because of the anticipation of pain during blood collection or because of fear of seeing blood.

### Concerns and Doubts About HIVST Use by Key Populations

**Figure 1:**
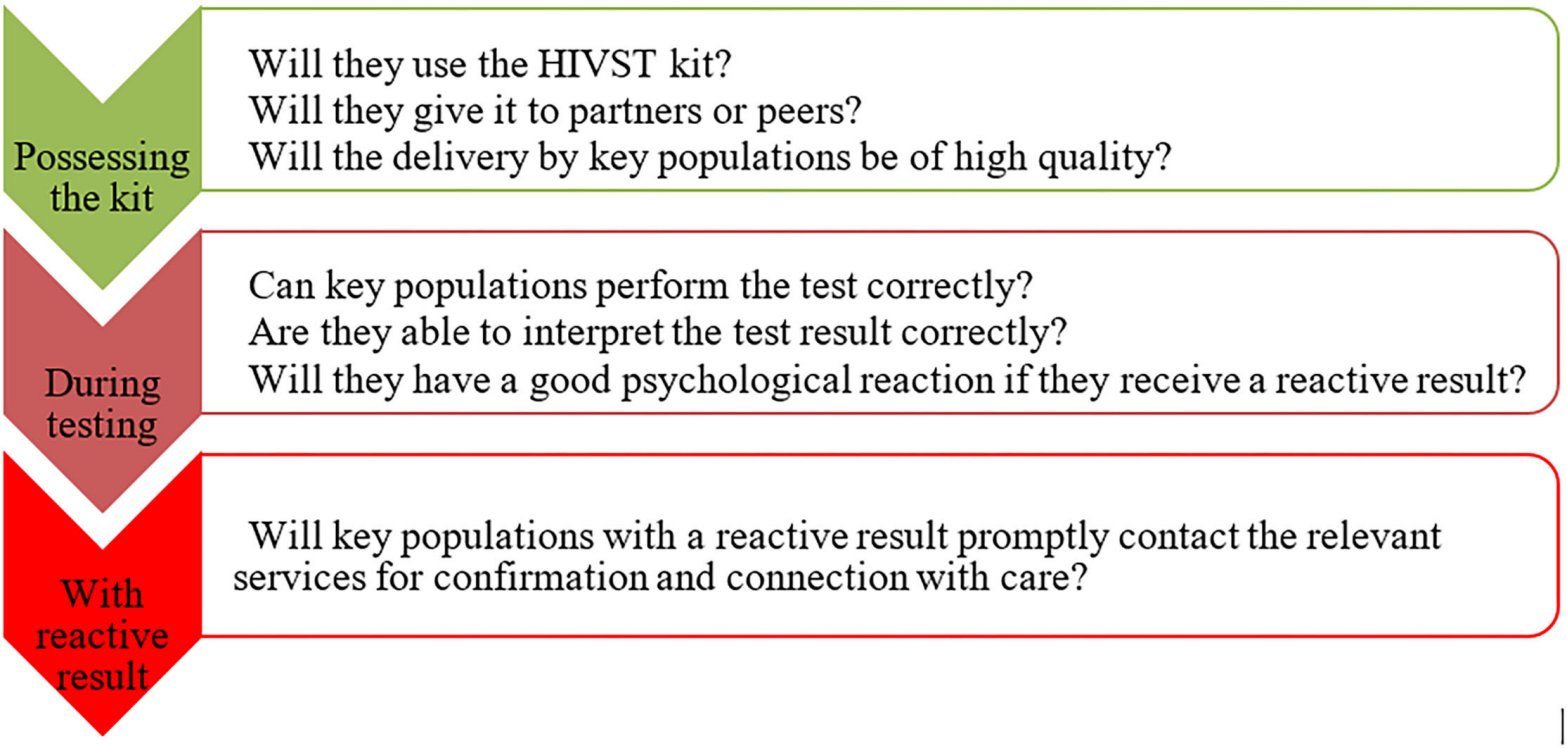
Participants' main question concerning the use of HIVST by key populations.

Though most participants are enthusiastic, have positive perceptions of HIVST and present a favorable attitude toward its introduction, interviewees in all three countries have questions, doubts or concerns, most of them related to the abilities of members of key populations (Figure 1). These stakeholders' concerns are summarized in five questions: HIVST kit retention, key populations' capacities to distribute HIVST kit, to perform it correctly, to manage themselves in case of reactive result. Finally, they wondering how to measure usual HIV testing indicators.

#### With the HIVST Kit in Hand and Without Supervision, Will Key Populations Use It?

Within the ATLAS framework, in primary distribution, the HIVST kit is given to users for their own use, with or without a provider's assistance. They also benefit from counseling and audio-visual or written support to them help with the test and with connecting with care. Some respondents have doubts that the HIVST kit will actually be used without provider assistance. These doubts were most often expressed by participants in Côte d'Ivoire and Senegal. According to these stakeholders, the fear of discovering a reactive HIVST result can hinder HIVST use. Use might also be low in *situations* where the user has not fully understood the procedures for performing the test or where the user is not confident and has doubts about his or her ability to perform it correctly.

Even when the distribution is done well, they say to themselves that they can't hold that because they will be alone at home; open it, put the tube, put the other tube, take something, take the saliva from the mouth, put it in the diluent and then read the result. It's too long compared to putting a finger on the Determine (traditional rapid HIV tests) or the Stat-Pak and then they read the result (Field coordinator, Côte d'Ivoire).

These doubts are more substantial regarding PWuID, as respondents feared that when performing HIVST, users might not have the full mental capacity to comply with HIVST instructions.

#### Once They Have HIVST Kits, Will Key Populations, Without Supervision, Ensure Secondary Distribution?

For secondary distribution, one or more HIVST kits are given to identified key population members to be redistributed to their partners, peers or clients. Some stakeholders expressed doubts, especially in Côte d'Ivoire, regarding the ability of key population members to redistribute HIVST kits. From their perspective, to redistribute HIVST, it is necessary to have good knowledge of HIV and basic HIV counseling information. They worried that some primary contacts might not be able to assimilate all the information delivered during primary distribution (particularly regarding how to perform the test and the importance of confirmatory testing) and share it correctly with their secondary contacts.

My main concern is about secondary targets. It won't be all the FSW or all the MSM who will receive HIVST kits who will be able to really pass all the information back to secondary users (Hotline manager, Côte d'Ivoire).

For HIVST, if an FSW gives the kit to her partner, she will briefly explain to him the procedure and all that, but I mean she doesn't have all the information to manage the result announcement and then the partner will have to face his result alone (National NGO stakeholder, Côte d'Ivoire).

Members of key populations that are not confident may avoid raising the subject with partners or peers to whom HIVST kits should be provided. Doubts about the technical capacity to ensure correct delivery of HIVST to partners were more pronounced for FSW and PWuID than MSM.

In a context of prevalent HIV/AIDS stigma, where having or offering an HIVST kit can be associated with being a PLHIV, some participants feared that FSW's or MSM's willingness to redistribute an HIVST kit to their regular partner might be limited.

She (FSW) refuses to give it to her boyfriend she is dating, for fear that he might suspect her. I'm going out to help my family, I'm not doing it for anything else; I don't want to have another problem there. What I'm doing here is also a concern, so I don't want to create more problems (FSW social support provider, Senegal).

Some respondents expressed concerns regarding the ability to redistribute HIVST kits of individuals who face social and economic vulnerabilities. This would potentially be the case among FSW who fear their partners' reactions because they do not know that they engage in sex work or know that they engage in sex work but otherwise manage the FSW's money and have influence and authority over them by protecting them at sex work sites. Regarding clients, fear of losing them by openly discussing HIV may limit the willingness of FSW to redistribute HIVST kits to their clients.

The main difficulty I see is the boyfriend, the regular client and not the occasional client, because among these clients there is one who is not a client (.) who is the partner, who is the concubine, who is the husband, has power that you can't even imagine. When you agree to have sex with a man without a condom, because you are so weak that you must negotiate the use of a condom, I ask myself the question: will that person have the audacity or the ability to get his partner to take the test (Program manager, International NGO, Senegal)?

Because it can put them in a dangerous situation (FSW) in terms of their own status and if they give out a self-test to their clients; they will think that they are positive and that will create a problem in their business and it can expose them as well (Researcher, Côte d'Ivoire).

The fear of partner misreactions could also limit the willingness of MSM to offer HIVST to their partners.

The situation could be more complicated for members of key populations living with HIV. Some respondents expressed doubts that HIVST kit redistribution would be optimal in such a situation, given the low level of HIV status disclosure among couples in these countries.

The problem is disclosure of HIV status. How do I bring a self-test home, which I can give to my partner, who is not informed of my status? What question is he going to ask me, how do I answer this question: ‘Where are you coming from? What did you do in this facility to get a self-test? What could you say about me there?' I think that's what might be blocking the thing (Medical doctor, PLHIV care, Côte d'Ivoire).

PLHIV often do not share their status with their partner; first difficulty. If you don't share the status with your partner, how can you come and tell him or her to do the self-test? It's quite a job; people are reluctant to share their status. In this respect alone, others will not adhere because they are not aware of the information (HIV focal point, public office, Senegal).

According to these respondents, the consequences of such situations would be the retention of the kits by key populations, the dissimulation of the nature of the test to the partner, a poor quality of distribution that could lead to non-use because the user would not understand the message or feel unable to perform the test, or a test that is performed without correctly following the instructions for use.

#### Will Users Be Able to Perform HIVST Correctly?

Although there are support tools for HIVST, including videos, a few respondents expressed doubts about the ability to perform the test and correctly interpret the results of members of key populations who are unable to read instructions.

If a person is illiterate, even though it has been translated into the national language with the video inside, it can be a barrier in any case. Maybe they're not going to do it well, maybe they're not going to interpret the results properly, maybe they don't know if they have the results, what about these results? Is it reactive? What must he/she do, and so on (Medical doctor, key population care, Senegal).

These doubts mainly relate to secondary distribution. According to the participants, these concerns are less important for MSM than for PWuID. Misuses of the test or reading errors could produce false results. This could have negative consequences, including the discrediting of HIVST, which could negatively influence key populations' adherence to this innovative strategy.

This means that people should not make mistakes in using the test. At this level, if the test is not well-performed, it can generate errors and doubt about its effectiveness, although the requirements have not been met (Medical doctor, key population care, Senegal).

Concerns that PWuID would not be able to perform HIVST properly were minimized by a peer educator and a key informant who has many years of experience providing various services to PWuID. They claimed that PWuID have the intellectual capacity to perform the test and would not be continuously under the effect of drugs.

#### Will Users Be Able to Self-Manage in the Case of a Reactive Result?

Referring to the usual strategies, where HIV test results are reported by a trained provider who has the appropriate tools and skills to do so, some participants expressed concerns about the reactions that members of key populations might have when confronted with a reactive HIVST result in a context in which they are alone.

Some people might find out their HIV status, be confused, be disoriented, be unable to make the right decision (Medical doctor, key populations care, Mali).

For these participants, counseling is one of the decision support tools that HIVST lacks, particularly when HIVST is administered at the secondary level by key populations rather than providers. They claim that without quality counseling, denial of results may be much greater than when using usual strategies.

We, we offer the classic test, and there are some positives even that are in denial. He knows his status and you know it. In spite of that, he denies it (Peer educator, MSM, Côte d'Ivoire).

On the basis of their experience with usual strategies, peer educators expressed some additional concerns about “losing” some positive people between HIV testing and care services. In Côte d'Ivoire and Mali in particular, interviewees questioned the strategy of systematic confirmation of reactive tests when key populations would not benefit from their support.

When the test is reactive, do they have the strength, the courage to go for a confirmatory test (Hotline manager, Côte d'Ivoire)?

Because it is precisely the person concerned who interprets the result! It is he himself who can go get confirmation. If he decides not to get confirmation, what we want to achieve, it's going to be really difficult to reach it (Stakeholder, public office, Mali).

However, some participants thought that, whether in the short-term or long-term, key populations with a reactive test would ultimately obtain confirmation of their results at some point.

#### How Will Their Work Be Acknowledged Without the Usual HIV Testing Indicators?

HIVST's unique feature is that it allows users to determine their HIV status privately, without the provider if they so desire. While respondents mentioned this as one of the strengths of this new strategy, they seemed to be somewhat disappointed with the lack of information about the HIV test results, both at the individual provider and program levels.

At the individual level, from the providers' perspective, without awareness of members of key populations' HIVST results, they cannot fully play their usual role in monitoring and supporting them.

Usually, providers want to have people's test results… The important thing is that in the end, either the person enters a process where he/she will be aware of his or her HIV-negative status and adopt safer behaviors, or the person is HIV-positive and the provider will fight to get him or her into care and have his or her viral load suppressed (Medical doctor, NGO responsible, Côte d'Ivoire).

On the other hand, across the 3 countries, there were lay providers who were rewarded by some NGOs based on their performance results. Such recognition is essentially related to the number of PLHIV that they have identified. Without any feedback on HIVST results from members of key populations, peer educators mentioned that the assessment of their individual performance could take into account the number of PLHIV identified through this new strategy.

Finally, at the program level, in all three countries, participants regretted that statistical data on their individual and programmatic efforts in providing HTS through HIVST would not be available, neither the numbers of key population members reached and tested, nor the number of HIV positives detected and linked to care services. This issue was important mainly for respondents from AIDS councils and other NGO stakeholders.

How to capture the impact, I will say the national result? It's true that we can rely on the fact that, if we see that at the national level the numbers of positives are increasing, we will certainly say that it is HIVST that has brought something. But I mean, the difficulty is to know the real impact, to be able to measure the impact on the result (stakeholder, government office, Côte d'Ivoire).

Among the five matters of concern expressed by stakeholders, four are related to the ability of users to perform HIVST, while one is related to data management in the health system.

### The Participants' Suggestions in Relation to the Perceived Abilities of the Members of the Key Populations to Use and Distribute HIVST

In response to their fears and doubts about HIVST use by key populations, some participants considered that the monitoring of HIVST kits should be more active. They proposed diverse solutions and complementary interventions: a physical support to key population who need it, follow up of HIVST distributed, reference of key populations with reactive results to lay providers for test confirmation, and more communication on HIVST at the national level.

To respondents, providing direct support through counseling to people who have been given an HIVST kit until the testing process, may be useful, especially for PWuID. According to them, this would help to ensure high test quality and psychological support for users with a reactive test result.

Additionally, with the aims of helping members of key populations perform the test, providing them with moral or psychological support in the case of a reactive test result, and supporting them in accessing confirmatory testing and care services, some participants suggested that HIVST providers should perform post distribution follow-up with users and secondary providers.

It's up to the community-based providers to exert more effort, to really get involved in the task. It's not to track people who have a reactive result but to do more listening to look for possibilities of feedback (on test results). For example, a provider who gives HIVST kits to an MSM group, to go (after) and ask “Do you have any problem?” to try to get some feedback so these reactive cases do not escape care services (Medical doctor, NGO stakeholder, Mali).

This is already done by some MSM and FSW peer educators who took part in the survey.

Anyway, I call them with my other phone number because I have a professional number. So I always call people on that, and if I deliver them (HIVST kits), there are people who call me and there are people I call back. So this number is always available (Peer educator, MSM, Mali).

To minimize the fear of stigma related to visiting health facilities, some participants suggest that key populations wanting to do so should be given the opportunity to present to lay providers who are already performing usual testing for confirmation of reactive results.

But I think there is some complicity between key populations and lay providers; and the level of confidence between them is higher than between key populations and health workers. So if possible (we should) emphasize much more that confirmation of the status of the person (should be done) through the lay provider who is already able to do HIV testing to confirm the status of the person (Medical doctor, NGO stakeholder, Mali).

He received the HIVST, for example, he takes the test and then despite having taken the test, he still doesn't want to go to a center for confirmation. A peer can go to him/her if he/she gives us the opportunity to touch him/her so that we can do the confirmation test (Hotline manager, Côte d'Ivoire).

Finally, according to stakeholders, more communication on HIVST at the national level is needed. This would (1) inform people more widely about HIVST and empower those in need to seek HIVST kits, (2) facilitate the task of the providers, as potential users would be more informed and trained in the use of HIVST beforehand, and, (3) in the context of secondary distribution, catalyze communication on HIV and testing within couples. Social networks have been proposed for promoting HIVST among key populations.

Everybody without exception, whether it's MSM, whether it's FSW, today everybody is connected to social networks. Everyone has a phone. Everyone wants to keep up with the new technology. So it's a way to really reach a lot of people among key populations and also to make self-testing widely known (Stakeholder, public office, Mali).

## Discussion

In Côte d'Ivoire, Mali and Senegal, stakeholders who took part in the study did not have any experience with HIVST before ATLAS program implementation. Their perceptions and attitudes were a mixture of enthusiasm and reservations and are based on their specific knowledge and experience of their countries' contexts and key populations.

### The Stakeholders' Attitudes and Concerns vs. Those of Stakeholders in Other Contexts

Various studies on HIVST perceptions and attitudes have been conducted among stakeholders in many countries like Tanzania and South Africa and have also found favorable attitudes toward HIVST (24–28). Our findings show that even in a context with lower HIV prevalence, such as the study context, there is enthusiasm about the introduction of HIVST for most at-risk populations. The motivations for integrating HIVST are both operational (ease of use, time savings, reduced transport costs, complementarity to usual strategies, relieving congestion in health facilities) and social or health-related (stigma reduction, anonymity and confidentiality, user empowerment, ability to reach hidden populations). However, as an innovative strategy that has never been implemented on a wide scale in these countries, HIVST raises questions, doubts and fears, which were also described in other perception analyses. In Southern Africa and elsewhere, authors have described informants' reluctance to integrate HIVST, which is mainly motivated by the lack of counseling (24–26, 29) doubts about the reliability of the results due to users' inability to perform the test themselves (24, 25), and fear that the link to care may be weak without HTS provider involvement (24, 26). This favorable attitude is a key factor for the introduction of HIVST in these countries, while ensuring that the concerns of the stakeholders are addressed.

### Maintaining Confidentiality and Doubts About Access to Care and Support Services

Perceptions and attitudes in favor of HIVST in our study were mainly related to confidentiality and anonymity. These are the primary motivations for HIVST acceptance found in other perception studies like Ethiopia and South Africa (25, 30, 31). HIVST makes it possible to bypass health facilities or community-based organizations, reducing the risk of stigmatization (24, 30). It improves the provision of HTS for people who are afraid of attending health facilities or who may fear unwanted disclosure of their HIV status (25). This is relevant in the West African context, where PLHIV and key populations are even more stigmatized than in countries with a higher HIV prevalence (2, 18, 31). A pilot study in Senegal found that HIVST is an effective strategy for reaching key populations who have never been tested or who are reluctant to be tested (32). However, the observed perceptions that some subcategories of key populations, such as clandestine FSWs or hidden MSM, would be more concerned than others about HIVST seem to be little discussed in the published literature.

In the context of high stigmatization of key populations, doubts about their willingness to connect with care were found in this study. Connecting with confirmatory and care services following a reactive HIVST result is perceived as a challenge in almost all studies (24, 27, 33, 34). However, according to the WHO, people who used HIVST have the same link-to-care practices as those tested with providers' support (18). A previous pilot study in Senegal found that 57% of key population members with a reactive result used confirmatory services (32). This rate is higher than that for home testing followed by referral by a provider (35, 36). These findings should be used to promote HIVST in the countries.

### Perceived Empowerment but Little Trust in the User

The potential autonomy and empowerment afforded by HIVST, as foreseen by stakeholders in our study, has been described as a favorable factor for HIVST integration into HIV testing strategies (34). In South Africa, these were perceived as the main benefits of HIVST by women, whereas men preferred HIVST due to its convenience and efficiency (37). As part of index testing, HIVST contributes to empowering women who are HIV-positive to manage their health (38). Stakeholders' perception of the user as both a beneficiary and an actor when engaged in secondary distribution contributes to key population empowerment by HIVST, an aspect that has not been highlighted in previous studies outside of the study context. Reasons for users' low capacity to perform HIVST themselves have been analyzed in other contexts. A study consisting of video surveillance of unsupervised HIVST in Kenya, Malawi and South Africa showed that the main difficulties were related to the collection of biological samples and the interpretation of the results, as ≤ 25% of the participants correctly followed all the steps indicated (39). Misinterpretation of the results and difficulty understanding instructions were also noted by Wolyec et al. in the Democratic Republic of Congo (40). However, Asiimwe et al. have showed that unsupervised HIVST is feasible in a rural African context, with comparable results to supervised testing (41).

Illiteracy was described as a potential barrier to HIVST uptake in Africa (24, 34). Considering the analyses that highlighted the inadequacy of the manufacturer's instructions to support correct performance of HIVST, especially by illiterate people, support tools were developed through cognitive and reiterative tests within the ATLAS program to adapt them to the implementation countries. An assessment found that, without the manufacturer's instructions, these adapted tools were sufficient to allow users to perform HIVST correctly (42). Stakeholders' inadequate knowledge about these preparatory procedures may have influenced their perceptions of this aspect, which can be more deeply analyzed after effective HIVST distribution in the implemented countries.

HIVST reticence was more pronounced regarding secondary distribution due to the absence of provider support throughout the process. However, stakeholders perceived HIVST secondary distribution as the best strategy for the hardest-to-reach key populations, thus accelerating the achievement of the first 90. Uncertainties about the ability of primary contacts to assure good counseling to end users have also been described elsewhere (43). However, secondary distribution to partners has been carried out successfully in the context of couple testing, health workers (43, 44) and among MSM (45, 46). Gender norms and power imbalances could negatively impact the ability of a woman to propose HIVST to her male partner, as mentioned in our study regarding FSW and as observed in other studies among pregnant women (33). Providing tips to primary contacts so that they have the necessary capacity to inform, negotiate and offer the HIVST kit remains essential for secondary distribution. This requires individual discussion between the providers and the primary contacts, not only to assess their HIVST knowledge and skills but also to discuss the relationship between the primary contact and the end user and provide adapted instructions for effective delivery without major adverse events.

Among the three key populations, stakeholders expressed the greatest concern for PWuID, for whom the ability to use HIVST is almost absent in the literature, to our knowledge. Further analysis is needed to understand if this concern is expressed by stakeholders who usually work with this key population and whose opinion is based on experience or if it relies on social representations focused on subcategories of PWuID, such as people permanently on heroin. This practice is uncommon in our context.

In response to their own concerns about the capacity of key populations to use HIVST, study participants made suggestions. These include (a) providing overall communication about HIVST to the general population, (b) ensuring direct assistance to HIVST users or follow-up after kit delivery, and (c) ensuring that there are close links between key populations and providers to whom they can come for confirmation and care if they so desire. Strengthening communication as a strategy for raising awareness, promoting HIVST and creating demand was also recommended by stakeholders in Haiti and Rwanda (33, 47). Such communication could facilitate both primary and secondary HIVST distribution. However, while support for testing and care by lay providers is useful, the requirement for direct assistance or systematic monitoring of kit distribution could be counterproductive. It could reduce the privacy offered by HIVST and users' autonomy, recognized by most study participants as a major advantage of this strategy. In this regard, the definition of support interventions that do not infringe on users' autonomy may depend on previous contexts for HTS and relationships between key population communities and health teams or peers educators and should be adapted at the national or site level.

### Overall Trends

Little difference was observed across the three countries, but all countries showed slight differences compared to the study results obtained from Eastern and Southern Africa. The importance of HIV stigma was highlighted by stakeholders, who pointed to the risk that HIVST users with a reactive result could be stigmatized within communities already stigmatized for “deviant behavior” as key populations: HIV stigma is considered by stakeholders as a barrier to HIVST uptake. Additionally, the importance of HIV stigma may explain why disclosure of HIV status by users to their partners is considered a main barrier to secondary distribution. Finally, the study results show that according to stakeholders, this determinant, which is unspecific to HIVST, may be a main barrier to HIVST efficiency. Stigma may also explain differences in issues identified by stakeholders in those countries compared to Eastern and Southern Africa.

Finally, although the study was focused on difficulties of HIVST integration for users, a crucial aspect was mentioned by stakeholders in all three countries. If HIVST protects users' anonymity, its use or the result of the test is not always known by providers. Therefore, and contrary to traditional HTS approaches, it is not possible to directly measure utilization or the positivity rate. It seems that providers feel they are losing power. In a context where international donors usually evaluate the efficiency of their programs using such quantitative indicators and where stakeholders are strongly encouraged to collect them, peer educators and program heads expressed trepidation regarding the assessment and recognition of their effort. To a certain degree, HIVST is a paradigm shift that requires the revision of evaluation tools and reflective exchanges among HTS stakeholders, program managers and funding institutions to overcome this potential obstacle to the promotion of HIVST based on user empowerment.

As found in other studies, these results suggest strongly the feasibility of HIVST in the study's context, where HIV prevalence is globally low, and key populations are highly stigmatized. Indeed, stakeholders are favorable for HIVST introduction in these countries, even if some reluctance has been expressed. These reserves should be minimized by providing data on the ability of “non-professional” and illiterate people of these countries, to self-test with ATLAS adapted tools, also in rural areas. HIVST must be part a strategy for key populations testing in these countries.

### Study Limitations

This qualitative study may be one of the first to provide information on the perceptions and attitudes of key HTS stakeholders in French-speaking West African countries. Participant selection in each country took into account interviewees' field experience and knowledge of HTS derived from various roles at several levels, in urban and rural areas. However, the study was conducted at the initiation of HIVST, and the collected perceptions were based on anticipation and may be influenced by social representations: they did not describe actual issues in the field. Stakeholders' perceptions may change during HIVST implementation. The results cannot be generalized to all HTS stakeholders in the three countries. Though these considerations do not correspond to the definition of a study limitation, we consider that it may be useful to emphasize that stakeholders' perceptions, which do not strictly reflect reality, must be considered for strategic introduction and integration of HIVST within the health system.

## Conclusion

In the three countries, HIVST is a strategy generating interest in improving key populations' access to HTS. Stakeholders' perceptions and attitudes are favorable to the introduction and integration of HIVST for several reasons. HIVST is considered to reduce stigma; preserve anonymity and confidentiality; reach key populations that do not access testing *via* the usual strategies; remove spatial barriers; save time for users and providers; and empower users with autonomy and responsibility. It is non-invasive and easy to use. Also HTS stakeholders have expressed concerns about users' ability to perform the test correctly; to ensure quality secondary distribution; to accept a reactive test result; and to use confirmation testing and care services. These results suggest strongly the feasibility of HIVST in the study's context. Providing to stakeholders, data on the ability of “non-professional” and illiterate people of their countries, to self-test could be useful.

## Data Availability Statement

The original contributions presented in the study are included in the article/Supplementary Material, further inquiries can be directed to the corresponding author/s.

## Ethics Statement

This study involving human participants was reviewed and approved by the WHO and the countries' Ethics Committees: WHO Ethical Research Committee (2019, August 7th, reference: ERC 0003181), National Ethics Committee for Life Sciences and Health of Côte d'Ivoire (2019, May 28th, reference: 049-19/MSHP/CNESVS-kp), Ethics Committee of the Faculty of Medicine and Pharmacy of the University of Bamako, Mali (2019, August 14th, reference: 2019/88/CE/FMPOS), and the National Ethics Committee for Health Research of Senegal (2019, July 26th, protocol SEN19/32). The research information sheet was read to respondents before each interview. For face-to-face interviews, all individuals signed a written consent form covering their participation and the audio recording. Oral consent was obtained from respondents who were interviewed by telephone.

## Author Contributions

OK-Z, AD, NR, and JL: conception or design of the work, interpretation of data for the work, drafting the work, and agreement to be accountable for all aspects of the work in ensuring that questions related to the accuracy or integrity of any part of the work are appropriately investigated and resolved. DP, SB, AV, and MM-G: contributions to the conception and design of the work, drafting of the work and revision for important intellectual content, and approval of the version to be published. AK, SS, SC, and SY: contribution to the data collection, drafting of the work, and revision for important intellectual content. All authors have read and approved the final version of the submitted manuscript.

## Funding

This work was supported by Unitaid (Grant No. 2018-23-ATLAS) with additional funding from Agence Française pour le Développement (AFD).

## Conflict of Interest

The authors declare that the research was conducted in the absence of any commercial or financial relationships that could be construed as a potential conflict of interest.

## Publisher's Note

All claims expressed in this article are solely those of the authors and do not necessarily represent those of their affiliated organizations, or those of the publisher, the editors and the reviewers. Any product that may be evaluated in this article, or claim that may be made by its manufacturer, is not guaranteed or endorsed by the publisher.
