# Supplementary material for: Enthusiasm for Introducing and Integrating HIV Self-Testing but Doubts About Users: A Baseline Qualitative Analysis of Key Stakeholders' Attitudes and Perceptions in Côte d'Ivoire, Mali and Senegal
Source: Front Public Health. 2021 Oct 18;9:653481. doi: 10.3389/fpubh.2021.653481 (PMC8558355; doi:10.3389/fpubh.2021.653481)
Supplement: Supplementary file 1 [file Table_1.DOCX]

**Composition of the ATLAS Team**

|  | **ATLAS Research Team** |
| --- | --- |
| Amani [Elvis Georges](https://www.notion.so/Elvis-Georges-Amani-d7395a112f144d2cb673123bae5c6329) | Programme PACCI, ANRS Research Site, Treichville University Hospital, Abidjan, Côte d'Ivoire. |
| [Badiane](https://www.notion.so/K-ba-Badiane-9447b415454a42e396b6fba69e7ee172) Kéba | Solthis, Sénégal |
| Bayac Céline | Solthis, France |
| [Bekelynck Anne](https://www.notion.so/Bekelynck-Anne-f6587a3b9a3c4ae5b5630907c0c986fa) | Programme PACCI, ANRS Research Site, Treichville University Hospital, Abidjan, Côte d'Ivoire |
| B[oily Marie-](https://www.notion.so/Boily-Marie-Claude-54b3fbd890bf46fbbf5b1e862cf604f0)C[laude](https://www.notion.so/Boily-Marie-Claude-54b3fbd890bf46fbbf5b1e862cf604f0) | Medical Research Council Centre for Global Infectious Disease Analysis, Department of Infectious Disease Epidemiology, Imperial College London, London, United Kingdom |
| Boye [Sokhna](https://www.notion.so/Sokhna-Boye-be416eba7a824a8c941ae700840b6795) | Centre Population et Développement, Institut de Recherche pour le Développement, Université Paris Descartes, Inserm, Paris, France |
| [Breton Guillaume](https://www.notion.so/Breton-Guillaume-16d3581e74dc45c4a5c0ca31a5ac50ea) | Solthis, Paris, France |
| [d'Elbée Marc](https://www.notion.so/d-Elb-e-Marc-b776f2ae8ea34685bd43aed0e36872d5) | Department of Global Health and Development, Faculty of Public Health and Policy, London School of Hygiene and Tropical Medicine, London, UK |
| D[esclaux Alice](https://www.notion.so/Desclaux-Alice-7241657b7fff4d009ef03c84afd9f97d) | Institut de Recherche pour le Développement, Transvihmi (UMI 233 IRD, 1175 INSERM, Montpellier University), Montpellier, France/CRCF, Dakar, Sénégal |
| [Desgrées du Loû Annabel](https://www.notion.so/Desgree-du-Lou-Annabel-ba07e466040e4407b582f88996d29f5b) | Centre Population et Développement, Institut de Recherche pour le Développement, Université Paris Descartes, Inserm, Paris, France |
| Diop Papa Moussa | Solthis, Sénégal |
| Ehui Eboi | Directeur Coordonnateur, PNLS |
| Graham Medley | Department of Global Health and Development, Faculty of Public Health and Policy, London School of Hygiene and Tropical Medicine, London, UK |
| [Jean Kévin](https://www.notion.so/Jean-K-vin-3a288b7aeb8b4d71a3adf60301a95325) | Laboratoire MESuRS, Conservatoire National des Arts et Métiers, Paris, France |
| Keita [Abdelaye](https://www.notion.so/Abdelaye-Keita-6c1586ce65a742509d2e5adcacb082a8) | Institut National de Recherche en Santé Publique, Bamako, Mali |
| [Kouassi Kra](https://www.notion.so/Ars-ne-Kouassi-Kra-21bd2bbd428343ba9256254cf9016f44) Arsène | Centre Population et Développement, Institut de Recherche pour le Développement, Université Paris Descartes, Inserm, Paris, France |
| [Ky-Zerbo Odette](https://www.notion.so/Ky-zerbo-Odette-fbe49e1a096547ba90bbd1b76181034b) | TransVIHMI, IRD, Université de Montpellier, INSERM |
| L[armarange](https://www.notion.so/Larmarange-Joseph-1dcdf20ef7ba4599b2c2bebcb6113971) J[oseph](https://www.notion.so/Larmarange-Joseph-1dcdf20ef7ba4599b2c2bebcb6113971) | Centre Population et Développement, Institut de Recherche pour le Développement, Université Paris Descartes, Inserm, Paris, France |
| [Maheu-Giroux Mathieu](https://www.notion.so/Maheu-Giroux-Mathieu-907fc88b747d4b17b1232b41f16ec5a6) | Department of Epidemiology, Biostatistics, and Occupational Health, School of Population and Global Health, McGill University, Montréal, QC, H3A 1A2, Canada |
| [Moh Raoul](https://www.notion.so/Moh-Raoul-bd729f67dd37479bb0ff89f3a7e53343) | 1. Programme PACCI, ANRS Research Site, Treichville University Hospital, Abidjan, Côte d'Ivoire.  2. Department of Infectious and Tropical Diseases, Treichville University Teaching Hospital, Abidjan, Côte d'Ivoire.  3. Medical School, University Felix Houphouet Boigny, Abidjan, Côte d'Ivoire |
| [Mosso Rosine](https://www.notion.so/Mosso-Rosine-12dade63b5d2418c9ddb4cec4570dc80) | ENSEA Ecole Nationale de Statistiques et d'Economie Appliquée, Abidjan, Côte d'Ivoire |
| [Ndour Cheikh Tidiane](https://www.notion.so/Pr-Ndour-Cheikh-Tidiane-e66b84ae4d0d495c8aa7bf8c91608ff0) | Division de Lutte contre le Sida et les IST, Ministère de la Santé et de l'Action Sociale Institut d'Hygiène Sociale, Dakar, Sénégal |
| [Paltiel David](https://www.notion.so/Paltiel-David-b264ed72dadd4d25870d38719ea82e39) | Yale School of Public Health, New Haven, CT, USA |
| [Pourette Dolorès](https://www.notion.so/Pourette-Dolor-s-36402926706145248de586073fc128ce) | Centre Population et Développement, Institut de Recherche pour le Développement, Université Paris Descartes, Inserm, Paris, France |
| [Rouveau Nicolas](https://www.notion.so/Rouveau-Nicolas-6ace639301f34853bd4b19dc681aaede) | Centre Population et Développement, Institut de Recherche pour le Développement, Université Paris Descartes, Inserm, Paris, France |
| [Silhol Romain](https://www.notion.so/Silhol-Romain-e2fe7bbd45b64ad7a1d354042c4926d2) | Medical Research Council Centre for Global Infectious Disease Analysis, Department of Infectious Disease Epidemiology, Imperial College London, London, United Kingdom |
| [Simo Fotso](https://www.notion.so/Arlette-Simo-Fotso-ceee998dafd74b3b935ac9059d4c82a2) Arlette | Centre Population et Développement, Institut de Recherche pour le Développement, Université Paris Descartes, Inserm, Paris, France |
| [Terris-Prestholt Fern](https://www.notion.so/Terris-Prestholt-Fern-eae075a74e004b6190a6cbf7d8054e26) | Department of Global Health and Development, Faculty of Public Health and Policy, London School of Hygiene and Tropical Medicine, London, UK |
| [Traore](https://www.notion.so/M-togara-Mohamed-Traore-94589a7530a24ed7aefd2d46d1bdf8b1) Métogara Mohamed | Solthis, Côte d'Ivoire |
|  | **Solthis coordination team** |
| Diallo Sanata | Solthis, Dakar, Sénégal |
| [Doumenc-Aïdara](https://www.notion.so/Cl-mence-Doumenc-A-dara-a71b566697864ffe9a227acca77af5d3) Clémence | Solthis, Dakar, Sénégal |
| [Geoffroy](https://www.notion.so/Olivier-Geoffroy-9ae6efdb6861466bb292024fe0d3a6d6) [Olivier](https://www.notion.so/Olivier-Geoffroy-9ae6efdb6861466bb292024fe0d3a6d6) | Solthis, Abidjan, Côte d'Ivoire |
| Kabemba [Odé Kanku](https://www.notion.so/Od-KANKU-KABEMBA-fdd3be902bd34ac6a17d71323502c7cc) | Solthis, Bamako, Mali |
| [Vautier Anthony](https://www.notion.so/Vautier-Anthony-23d8a704ce10434bb8ddf5b4d435ec88) | Solthis, Dakar, Sénégal |
|  | **Implementation in Cöte d’Ivoire** |
| [Abokon Armand](https://www.notion.so/Dr-Armand-ABOKON-705492b96e56435c801b4282d55a78c8) | Fondation Ariel Glaser, Côte d'Ivoire |
| [Anoma Camille](https://www.notion.so/Dr-Camille-ANOMA-384d3874b0ee4a2786eb8c051f325de6) | Espace Confiance, Côte d'Ivoire |
| [Diokouri](https://www.notion.so/Dr-Annie-DIOKOURI-9a1e77785b674bfbb4095a7fa7222428) [Annie](https://www.notion.so/Dr-Annie-DIOKOURI-9a1e77785b674bfbb4095a7fa7222428) | Fondation Ariel Glaser, Côte d'Ivoire |
| [Kouame Blaise](https://www.notion.so/Dr-Blaise-KOUAME-1e4513a6640f49eeadba700517276986) | Service Dépistage, PNLS |
| [Kouakou Venance](https://www.notion.so/Dr-Venance-KOUAKOU-b9c776f673924dd19a8c47e057b55d92) | Heartland Alliance, Côte d'Ivoire |
| [Koffi Odette](https://www.notion.so/KOFFI-Odette-d050f4d4459f4076be248a539220f6a6) | Aprosam, Côte d'Ivoire |
| Kpolo [Alain-Michel](https://www.notion.so/Alain-Michel-KPOLO-835d3fb5b88a48629613d8dd7338782f) | Ruban Rouge, Côte d'Ivoire |
| Sanogo [Abdoulaye](https://www.notion.so/Abdoulaye-SANOGO-2e85b004ca4546c19035008bc18e7db6) | Amprode Sahel, Mali |
| [Tety Josiane](https://www.notion.so/TETY-Josiane-e46cbc6a44924823ab0e5810c32971b5) | Blety, Côte d'Ivoire |
| [Traore Yacouba](https://www.notion.so/TRAORE-Yacouba-f95f844b8db9474893fd229908e5686d) | ORASUR, Côte d'Ivoire |
|  | **Implementation in Mali** |
| [Bagendabanga Jules](https://www.notion.so/Dr-Jules-Bagendabanga-6095d315dc4343beb4791df9c06ee2ff) | FHI 360, Mali |
| Berthé [Djelika](https://www.notion.so/Djelika-Berth-62c2001634444844b89051b0aca8d58b) | PSI, Mali |
| Diakite [Daouda](https://www.notion.so/Daouda-Diakite-a85242c23a8d4138b5950096301bf4ac) | Secrétariat Exécutif du Haut Conseil National de Lutte contre le Sida, Mali |
| Diakité [Mahamadou](https://www.notion.so/Mahamadou-DIAKITE-1db01b1f327c4db5a5127dd15447a1ae) | Danayaso, Mali |
| Diallo [Youssouf](https://www.notion.so/Youssouf-Diallo-86ab4828bb2446ff99be2dca808df663) | CSLS/MSHP |
| [Daouda Minta](https://www.notion.so/Prof-Minta-Daouda-bebbe7a5ae9a444296c87a54d625b203) | Comité scientifique VIH |
| [Hessou Septime](https://www.notion.so/Dr-Septime-Hessou-65d8bdedbb044d78820f38c7cf13310f) | Plan Mali |
| Kanambaye [Saidou](https://www.notion.so/Saidou-Kanambaye-b1b71418cf57498b9c2f9bc600294897) | PSI, Mali |
| [Kanoute Abdul Karim](https://www.notion.so/Kanoute-Abdul-Karim-35b65acd9afb4cba9b49f9ec224488fe) | Plan Mali |
| [Keita Dembele Bintou](https://www.notion.so/Dr-Dembele-Bintou-Keita-e2e16458a090479a97095c2a9ee99e96) | Arcad-Sida, Mali |
| [Koné Dramane](https://www.notion.so/Dr-Dramane-Kon-6377bfbc3e5b4fe39cfbc854011b51d7) | Secrétariat Exécutif du Haut Conseil National de Lutte contre le Sida, Mali |
| Koné [Mariam](https://www.notion.so/Mariam-Kon-50ffd9a9bb6a4a949cbe4bb106fd9c27) | AKS, Mali |
| [Maiga Almoustapha](https://www.notion.so/Maiga-Almoustapha-8dd26e801081496b825e055c5b189b2c) | Comité scientifique VIH |
| [Nouhoum Telly](https://www.notion.so/Dr-Telly-Nouhoum-fb6c2a5b8b05466cad61530e3cabad35) | CSLS/MSHP |
| [Saran Keita Aminata](https://www.notion.so/Dr-KEITA-Aminata-Saran-2eb414403cfe45f3b2677c053ead66fe) | Soutoura, Mali |
| Sidibé [Fadiala](https://www.notion.so/Fadiala-Sidib-da56f184c8c44e96b7d0a0ec680da426) | Soutoura, Mali |
| Tall [Madani](https://www.notion.so/Madani-Tall-ca7f54d7ebb2481e8dc494cd75059dc0) | FHI 360, Mali |
| [Yattassaye Camara Adam](https://www.notion.so/Dr-Camara-Adam-Yattassaye-ab44119c6e34408d9de404f33bec50d1) | Arcad-Sida, Mali |
|  | **Implementation in Senegal** |
| [Bâ Idrissa](https://www.notion.so/Dr-Idrissa-B-911a7d7b6b3745d59f64344f35717ad8) | CEPIAD, Sénégal |
| [Diallo Papa Amadou Niang](https://www.notion.so/Dr-Papa-Amadou-Niang-293bf2ecfc464c3b84df949c71f0d31c) | CNLS, Sénégal |
| [Fall Fatou](https://www.notion.so/Dr-Fatou-Fall-7d2d7ed8ae78469eb2efd53b494f0704) | DLSI, Ministère de la Santé et de l'action sociale, Sénégal |
| [Guèye NDèye Fatou NGom](https://www.notion.so/Dr-ND-ye-Fatou-NGom-Gu-ye-c3161c72b7d9433eaa1b02cc79a9f8c7) | CTA, Sénégal |
| Ndiaye [Sidy Mokhtar](https://www.notion.so/Sidy-Mokhtar-NDiaye-d470d2be528b46fca7779d12cb4ac710) | Enda Santé, Sénégal |
| [Niang Alassane Moussa](https://www.notion.so/Dr-Alassane-Moussa-Niang-c52bafcebb69432587c1607a79e0f1d5) | DLSI, Ministère de la Santé et de l'action sociale, Sénégal |
| [Samba Oumar](https://www.notion.so/Dr-Oumar-Samba-df3d5f1bb0e641e5803e39a008b8cf64) | CEPIAD, Sénégal |
| [Thiam Safiatou](https://www.notion.so/Dr-Safiatou-Thiam-fbca1b25bd7b4f29baa0c7bbf08b03e9) | CNLS, Sénégal |
| Turpin [Nguissali M.E.](https://www.notion.so/Nguissali-M-E-Turpin-3fe470426b784b56ad7dd877b40bbc8b) | Enda Santé, Sénégal |
|  | **Partners** |
| Bouaré [Seydou](https://www.notion.so/Seydou-Bouar-f3669d3cfacd4b77ab99da4cad9c7934) | Assistant de recherche, Mali |
| [Camara Cheick Sidi](https://www.notion.so/Cheick-Sidi-Camara-dda932a72bb046568190b9b5cb3e30c0) | Assistant de recherche, Mali |
| Kouadio [Brou Alexis](https://www.notion.so/Brou-Alexis-Kouadio-f467590129bf470ebcefec47e06cef47) | Assistant de recherche, Côte d'Ivoire |
| [Sarrassat Sophie](https://www.notion.so/Sarrassat-Sophie-c197183699454536a53ef3ad56c4cc82) | Centre for Maternal, Adolescent, Reproductive and Child Health, London School of Hygiene and Tropical Medicine, London, UK |
| Sow [Souleymane](https://www.notion.so/Souleymane-Sow-6ca1b6d006d6497a96d1e72b050824c7) | Assistant de recherche, Sénégal |
