# Supplementary material for: Enthusiasm for Introducing and Integrating HIV Self-Testing but Doubts About Users: A Baseline Qualitative Analysis of Key Stakeholders' Attitudes and Perceptions in Côte d'Ivoire, Mali and Senegal
Source: Front Public Health. 2021 Oct 18;9:653481. doi: 10.3389/fpubh.2021.653481 (PMC8558355; doi:10.3389/fpubh.2021.653481)
Supplement: Supplementary file 2 [file Table_2.DOCX]

**Indepth individual interview guide**

**Respondent identification number _____________________________**

**Description of the respondent**

Country

Locality

Respondent category

Marital status

Level of schooling

Profession

Structure

Approximate age

Sex

**Interviews**

| **Topic** | **Questions** |
| --- | --- |
| General | ∎ Tell us about the situation and evolution of the HIV epidemic in Côte d'Ivoire, Mali, Senegal (choose the country indicated)  ∎ Is the epidemic the same in all parts of the country? Are some regions more infected than others?  ∎ From your point of view, which groups are more affected (men/women, key populations/general population, children/youth/adults)? |
| Reaching the first 90 | ∎Comment do you appreciate the level of achievement of the first 90 in (COUNTRY: choose the country indicated]?  ∎ For you, what justifies this level of screening in your country?  ∎ Which populations have the least access to HIV testing? How do you explain this? |
| Perceptions of **ADVIH** as a screening strategy | ∎Do we have screening strategies implemented in [COUNTRY] for the general population; key populations? Limitations and benefits of each strategy?  ∎Que do you think about **ADVIH**? Have you tried it yourself? In general, does it remind you of other self-tests?  ∎Que do you think about the introduction of **ADVIH** as a screening strategy in the country (for/against, reasons...)? Among key populations?  ∎Avantage comparison of **ADVIH in** relation to other strategies |
| **ADVIH in the framework of the ATLAS project**: opportunities and obstacles to the introduction of **ADVIH and** its support system | ∎ From your point of view, what could lead key populations to accept **ADVIH (**try to find out whether the motivations are the same from one key population to another)?  ∎what might prevent them from doing so (i.e. whether barriers are the same across key populations)?  ∎could facilitate the implementation of this project in (COUNTRY) at the political? social? health level?  ∎Quelles are the difficulties that the project is likely to face at the political? social? health? level. |
| **ADVIH in the framework of the ATLAS project**: opportunities and obstacles in the country's associative and health system | ∎ could be the benefits of ADVIH for the health system? for NGOs and associations involved in the fight against HIV?  ∎ Could this be what is preventing a good supply of ADVIH in health facilities? By NGOs and associations?  ∎ To health workers and community actors involved in the delivery of ADVIH  ∎ how does the introduction of ADVIH make it easier for you to offer screening to key populations?  ∎ How does this make it difficult for you to offer screening to key populations? What could help you? |
| ADVIH in the ATLAS project: opportunities and obstacles specific to each population | ∎ In your opinion, to whom (which population) should ADVIH be offered as a priority? Why  ∎ is the population for whom the provision of ADVIH is easiest? What explains this?  ∎ What is the population in which dispensing is difficult? What explains this?  ∎ In your opinion, are there risks of abuse, and if so, what are they?  ∎Craignez do you have people being forced to take the test against their will? Under what circumstances?  ∎ If coercive practices exist, how can victims report them and protect themselves from them?  ∎Population key by key population: what are the difficulties that actors may encounter in offering HIV and AIDS to this population? Difficulties that the key population concerned may encounter in dispensing to peer? partners? What are the difficulties that the key population may encounter in the self-administration of the supervised test? and in the self-administration of the unsupervised test? |
| Perceptions of the support system (advice, green line, tools) | ∎ The project uses secondary providers of kits for key populations; who do you think can play this role in TS, MSM and UD settings (gender, age, profession)?  ∎ How do you appreciate the system in place to give you advice to people who carry out ADVIH: face-to-face advice, green line (relevance, ease of access, quality of advice/responses, effectiveness, sustainability)?  ∎ What do you suggest instead or in addition? |
| Link with care services | ∎ What do you think of the current referral system based on telephone counselling?  ∎ In your opinion, what can we do to help self-tested people who have a positive result to use the facilities for confirmation? care? |
| Recommended adjustments for KP | ∎ In your opinion, what information should be given to key populations prior to self-testing? And after the result (explore for both negative and positive results)?  ∎ Or could HIV-AIDS be most accessible for TS, MSM and UD? At what point should ADVIH be offered to these different populations? |
